# Salivary Lactoferrin Levels and Polymorphisms in Down Syndrome Individuals with Periodontitis

**DOI:** 10.3390/jcm14061815

**Published:** 2025-03-07

**Authors:** Lucía Sande López, Eliane García-Mato, Alicia de Coo, Raquel Cruz, Desireé Antequera, Pedro Diz, Eva Carro, Berta Rivas

**Affiliations:** 1 Medical-Surgical Dentistry Research Group, Health Research Institute of Santiago de Compostela, School of Medicine and Dentistry, Santiago de Compostela University, 15706 Santiago de Compostela, Spain; luciasandelopez@gmail.com (L.S.L.); eliane.garma@gmail.com (E.G.-M.); berta.rivas@usc.es (B.R.); 2 Grupo de Medicina Xenómica, Centro Singular de Investigación en Medicina Molecular y Enfermedades Crónicas (CIMUS), Universidade de Santiago de Compostela, 15782 Santiago de Compostela, Spain; alicia14coo@hotmail.com (A.d.C.); raquel.cruz@usc.es (R.C.); 3 Neurobiology of Alzheimer’s Disease Unit, Functional Unit for Research into Chronic Diseases, Instituto de Salud Carlos III, 28029 Madrid, Spain; 4 Network Centre for Biomedical Research in Neurodegenerative Diseases (CIBERNED), ISCIII, 29016 Madrid, Spain

**Keywords:** Down syndrome, periodontal disease, salivary biomarker, lactoferrin

## Abstract

**Background/Objectives**: Lactoferrin, a protein involved in the immune response, plays a significant role in the etiopathogenesis of periodontitis in the general population. This cross-sectional study aims to determine the salivary concentration of lactoferrin in Down syndrome individuals with periodontitis. **Methods**: A convenience cohort of 76 Down syndrome individuals was established, including 34 with periodontitis, 29 with gingivitis, and 13 with healthy gums. Unstimulated saliva samples were collected and processed to quantify the lactoferrin concentration using the Human Lactoferrin ELISA kit, the total protein concentration (bicinchoninic acid assay [BCA]) using the BCA Protein Assay Kit (Pierce, Rockford, IL, USA), and the lactoferrin/BCA ratio. Additionally, the Lf rs1126478 (140A/G in exon 2, Lys/Arg) genotypes were determined via PCR with restriction fragment length polymorphism (RFLP) analysis using the Earl enzyme. **Results**: The lactoferrin levels were comparable across patients with periodontitis, gingivitis, and healthy gums (median = 8.20, 6.57, and 7.61 µg/mL, respectively). There were no differences in the BCA levels between the three groups (median = 2.21, 3.17, and 2.08 µg/µL, respectively) nor in the lactoferrin/BCA ratios. The distribution of the Lf 140A/G polymorphism did not show differences concerning periodontal health status. **Conclusions**: In Down syndrome individuals, salivary lactoferrin and BCA levels are not influenced by the periodontal health condition. Additionally, no significant genetic associations were found with the rs1126478 polymorphism in Down syndrome individuals with and without periodontitis. Lactoferrin production in Down syndrome may not be upregulated in response to periodontal pathogens, which could be indicative of an immune system dysregulation contributing to the early onset and severity of periodontitis in this population.

## 1. Introduction

### 1.1. Down Syndrome and Periodontal Disease

Down syndrome (DS) is the most common chromosomal disorder, with an estimated prevalence of 1 case per 800 live births [1]. It is characterized by a variable degree of intellectual disability and a range of systemic issues, such as congenital heart defects, hematological disorders, and endocrinological dysfunctions [2,3]. DS also impacts craniofacial development, leading to a distinct phenotype. Common orofacial manifestations include macroglossia, dental malocclusions, delayed tooth eruption, and periodontal disease [4,5].

Individuals with DS have a higher prevalence of periodontal disease compared to the general population, with rates ranging from 58% to 96% in those under 35 years old [6]. However, several of the epidemiological studies supporting this claim are limited by significant methodological biases [7]. In DS patients, periodontitis typically appears early, is widespread, and progresses rapidly and severely [8]. According to the current classification of periodontal diseases, periodontitis in DS individuals would be classified as “periodontitis as a manifestation of a systemic disease or condition” [9].

Historically, the etiopathogenesis of periodontal disease in DS has been linked to local factors such as poor oral hygiene, macroglossia, abnormal dental morphology, gingival tissue abnormalities, and saliva characteristics [10]. However, recent studies have highlighted a multifactorial etiology, with immune system dysregulation playing a central role [11]. This dysregulation weakens the immune response to periodontal pathogens, leading to bacterial dysbiosis [12] and exaggerated local inflammatory responses [13], especially in genetically predisposed individuals [14].

### 1.2. Periodontitis and Salivary Lactoferrin

Salivary biomarkers are emerging as non-invasive tools for the early detection, risk assessment, and monitoring of periodontal disease treatment [15]. Advances in molecular biology—particularly proteomics and metabolomics—have enabled the identification of numerous salivary biomarkers of periodontal disease. These include tetraspanins (CD9 and CD81), soluble urokinase plasminogen activator receptor (suPAR), galectin-1, matrix metalloproteinase 9 (MMP-9), S100 calcium-binding protein A8 (S100A8), lactate dehydrogenase (LDH), aspartate aminotransferase (AST), and cytokines (IL-1β, IL-6, and TNF-α), which reflect the complex pathophysiology of periodontitis [15,16].

One promising biomarker for periodontal disease is lactoferrin, a glycoprotein from the transferrin family found in saliva and other bodily fluids. It is synthesized by exocrine glands and neutrophils in response to infection and inflammation [17]. Lactoferrin is an iron-binding protein with antimicrobial activity against periodontopathogenic bacteria such as *Aggregatibacter actinomycetemcomitans*, *Porphyromonas gingivalis*, and *Prevotella intermedia* [18]. It also has immunomodulatory properties, including the inhibition of pro-inflammatory cytokine production, thereby reducing gingival inflammation and preventing alveolar bone destruction [19].

In a case-control study, it was shown that in individuals with chronic periodontitis, salivary lactoferrin concentrations were significantly correlated with bleeding on probing and the number of sites with a probing pocket depth ≥ 6 mm [20]. In another recently published case-control study, Ramenzoni et al. [21] demonstrated that in individuals with Stage III Grade B chronic periodontitis, the concentration of lactoferrin in stimulated saliva can be up to three times higher compared to periodontally healthy individuals (median [IQR]: 28.5 [3.5] µg/mL versus 9.6 [1.8] µg/mL). Consequently, although no systematic review with meta-analysis on lactoferrin and periodontitis has been published to date, it has been suggested that salivary lactoferrin may be a reliable biomarker for periodontal disease [21].

Lactoferrin is encoded by the LTF gene located on chromosome 3p21.3. A missense polymorphism in exon 1, specifically p.Lys47Arg (GCC > ACC), results in a substitution of adenine (A) for guanine (G) at the rs1126478 marker, leading to an amino acid change from Lysine to Arginine. Some functional genetic variants of LTF have been associated with an increased susceptibility to periodontal disease, with a lower frequency of AA alleles and a predominance of GG alleles observed in patients with chronic periodontitis [22,23].

In individuals with DS, periodontitis is highly prevalent, manifests early, and follows an aggressive course. This study aims to determine whether individuals with DS and periodontal disease exhibit increased salivary lactoferrin levels and/or a predominant lactoferrin polymorphism compared to individuals with healthy gingiva. We hypothesize that the absence of significant differences would provide additional evidence supporting the immune dysregulation observed in DS, in which neutrophil dysfunction plays a pivotal role.

## 2. Materials and Methods

### 2.1. Participants

A convenience sample of 76 individuals, both male and female, aged over 18 years, with Down syndrome (DS) was recruited. All participants were regular attendees at special education centers in Santiago de Compostela, Lugo, and Madrid (Spain). Clinical assessments and sample collection were carried out between December 2022 and November 2023.

Eligibility criteria included a genetically confirmed diagnosis of DS, adequate cooperation to undergo clinical examination and sample collection, the absence of behaviors that could predispose to periodontal disease such as smoking, systemic diseases (e.g., diabetes and autoimmune diseases), the use of medications in the past month that could alter the inflammatory response (e.g., nonsteroidal anti-inflammatory drugs, corticosteroids, or immunomodulatory drugs), and provision of written informed consent by each participant or their legal representative. The study protocol was approved by the Research Ethics Committee of Santiago-Lugo (Xunta de Galicia; reference 2018/510, Spain).

### 2.2. Periodontal Diagnosis

To minimize the duration of the examination, it was restricted to the six “Ramfjord teeth” [24]. The probing pocket depth, bleeding on probing, and clinical attachment loss were assessed for each of these teeth. Measurements were performed on all four surfaces of each of the aforementioned teeth (buccal, palatal/lingual, mesial, and distal). In cases where any of the Ramfjord teeth were missing, the adjacent tooth in the same sextant was used instead.

Regarding gingival health status, individuals were classified as clinically healthy in the absence of clinical periodontal inflammation, either on an anatomically intact periodontium or a reduced periodontium [25]. Gingivitis was defined as a nonspecific inflammatory response to an accumulation of bacterial plaque, confined to the gingival tissue, without underlying destruction of the supporting tissues [25]. According to the criteria from the “2017 World Workshop on the Classification of Periodontal and Peri-Implant Diseases and Conditions”, periodontitis was defined as clinical attachment loss of ≥3 mm on the buccal surface, with probing depths > 3 mm on two or more teeth, or clinical attachment loss on the interdental area of two or more non-adjacent teeth [26,27].

### 2.3. Saliva Collection and Analysis

Unstimulated saliva samples were collected and processed from all participants as previously described [28]. The participants were instructed to refrain from eating, drinking, or performing oral hygiene procedures for at least 3 h prior to sample collection. Saliva samples (~0.5 mL) were collected in duplicate and stored frozen until processing.

One saliva sample from each participant was used to quantify protein and lactoferrin concentrations. The samples were centrifuged at 1000 rpm for 10 min at 4 °C, and the supernatants were aliquoted into polypropylene tubes containing a protease inhibitor cocktail (Roche Diagnostics, Mannheim, Germany). These aliquots were stored at −80 °C until further analytical processing.

The total protein concentration in the saliva samples was analyzed using a bicinchoninic acid (BCA) protein assay kit (Pierce, Rockford, IL, USA) following the manufacturer’s instructions. Lactoferrin levels in saliva samples were quantified using enzyme-linked immunosorbent assays (ELISAs) with the Human Lactoferrin ELISA Kit (FineTest, Wuhan, China), according to the manufacturer’s protocol. The intra-assay coefficients of variation ranged from 4.7% to 6.0%, the inter-assay coefficients ranged from 4.6% to 5.3%, and the lower detection limit was 0.3 ng/mL. The ratio of lactoferrin to BCA was referred to as the “ratio” and expressed as a percentage.

The second saliva sample from each participant was used to determine the genotype frequency for the lactoferrin marker rs1126478 (T/C). Genotyping and quality control procedures have been previously described in detail [14]. Briefly, DNA was extracted from unstimulated saliva samples and genotyped using the Axiom Spain Biobank Array (Thermo Fisher Scientific, Waltham, MA, USA) at the National Genotyping Center (CeGen-ISCIII), following the manufacturer’s protocol. The Axiom Spain Biobank Array is a panel specifically designed for the Spanish population, containing 757,836 markers, including the lactoferrin single-nucleotide polymorphism rs1126478. The results were compared to those of a control population of unrelated individuals from across Spain with a healthy periodontal condition, sourced from the “Banco Nacional de ADN-Instituto de Salud Carlos III” (BNADN-ISCIII; University of Salamanca, Spain, www.banco adn.org, accessed on 6 February 2025).

Due to primarily methodological reasons (insufficient or contaminated salivary samples), the determination of BCA and lactoferrin levels, as well as the genotype frequencies for the rs1126478 marker, could not be completed for all individuals in the study group (Figure S1).

### 2.4. Statistical Analysis

Statistical analyses were conducted using R software version 4.3.3 (R Development Core, Vienna, Austria). To identify the most appropriate statistical test for comparing lactoferrin, BCA, and the lactoferrin/BCA ratio across the three participant groups (DS with periodontal health, gingivitis, and periodontitis), normality was assessed using the Anderson–Darling test, and homoscedasticity was evaluated with the Fligner–Killeen test. As the *p*-values from both tests were below the significance level (α = 0.05), the null hypotheses of normality and homoscedasticity were rejected, and the Kruskal–Wallis test was applied.

Pearson’s chi-squared test was used to evaluate the distribution of lactoferrin rs1126478 genotypes and allele frequencies among the subgroups. In cases where the number of observations was fewer than 5, Fisher’s exact test was employed. The association between lactoferrin genotypes and periodontal disease was assessed using logistic regression, with case/control status as the dependent variable. Gender and age were included as covariates to adjust for potential confounding effects. Statistical significance was defined as a *p*-value of less than 0.05.

## 3. Results

**Figure 1:**
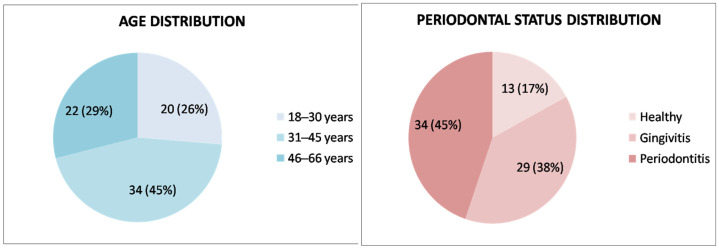
Age and periodontal health status distribution of the study group (*n* = 76).

Among the 76 participants in the study group, 46 were female (60.5%), and 30 were male (39.4%), with a mean age of 38.1 ± 12.2 years (range: 18–66 years). Regarding their periodontal health status, 13 (17.1%) were periodontally healthy, 29 (38.1%) had gingivitis, and 34 (44.7%) met the criteria for periodontitis (Figure 1).

The median lactoferrin levels were 7.43 µg/mL (Q1: 4.72 µg/mL; Q3: 10.86 µg/mL), while the median BCA levels were 265.68 µg/mL (Q1: 169.61 µg/mL; Q3: 407.83 µg/mL). The lactoferrin/BCA ratio had a median value of 2.57% (Q1: 1.78%; Q3: 4.52%). No statistically significant differences were observed between the periodontal health groups (periodontally healthy, gingivitis, and periodontitis) with respect to lactoferrin levels, BCA, or the lactoferrin/BCA ratio (Table 1).

The predominant allele for the rs1126478 marker (A/G) was the A allele, found in 70.8% of DS individuals and 68.9% of controls from the National DNA Bank. This pattern was consistent across codominant genotypes (AA), observed in 53.3% of DS individuals and 47.2% of controls, and particularly in recessive genotypes (AA + GA), found in 88.3% of DS individuals and 90.8% of controls. No significant differences were detected in the genotype or allele distribution of the rs1126478 marker, in any of its genetic forms of expression (codominant, recessive, or dominant), between DS individuals with and without periodontitis nor between the entire DS cohort and controls from the “National DNA Bank-Instituto de Salud Carlos III”. All these findings are summarized in Table 2.

## 4. Discussion

Recently, in a case-control study, we observed that salivary lactoferrin levels were significantly higher in DS cases (median: 7.43 µg/mL) compared to those observed in euploid controls (median: 4.44 µg/mL). Statistical analysis also showed that the DS group had a significantly higher median salivary protein concentration (265.68 µg/mL) than the control group (183.43 µg/mL) [29]. This led to the hypothesis that the high prevalence of periodontitis in individuals with DS might have contributed to these findings. However, in the current study, after analyzing unstimulated saliva samples using the enzyme-linked immunosorbent assay (ELISA), we found no statistically significant differences in the salivary levels of BCA, lactoferrin, or the lactoferrin/BCA ratio between DS individuals who were periodontally healthy, those with gingivitis, and those with periodontitis.

Numerous studies have confirmed elevated lactoferrin levels in chronic periodontitis patients compared to periodontally healthy individuals in the general population across various sample types, including unstimulated saliva [21,22], stimulated saliva [20,30,31], and gingival crevicular fluid [32,33,34,35,36]. Similar results have been reported in patients with aggressive periodontitis [37] and severe chronic periodontitis [38]. A key factor influencing these results is the sensitivity of the detection method, with most studies using enzyme-linked immunosorbent assay (ELISA) [21,22], although comparable outcomes have also been achieved using mass spectrometry [34,36].

To explain our results, we suggest that neutrophils, the most abundant cells in the gingival sulcus during the initiation and progression phases of periodontitis [33], may play a key role. It has been confirmed that salivary lactoferrin levels in areas of infection/inflammation increase primarily due to the recruitment of neutrophils [19]. In this regard, significantly higher lactoferrin concentrations have been detected in periodontal pockets compared to saliva and gingival crevicular fluid [21], with these levels correlating to pocket depth [39]. Furthermore, in patients with chronic periodontitis, a significant reduction in lactoferrin levels has been observed in samples taken from pockets with deeper probing depths following periodontal treatment, with lactoferrin concentrations in gingival crevicular fluid correlating with the type of treatment applied [33].

Recent studies have reported lower neutrophil counts in the blood of children with DS compared to age- and sex-matched healthy controls [40], but no such differences have been found in adults with DS versus non-syndromic controls [41]. In the general population, neutrophil concentrations in periodontal pockets increase significantly [39], but this finding has not been confirmed in individuals with DS. We suggest that neutrophil dysfunction in DS—characterized by a predominance of immature cells, reduced chemotaxis, and massive cytokine release—could play a role in the etiopathogenesis of periodontitis [42]. However, this dysfunction may not lead to neutrophil accumulation and, thus, does not result in a significant increase in salivary lactoferrin.

Another conclusion derived from this study is that lactoferrin is not a reliable biomarker for periodontal disease in individuals with DS. Among the salivary biomarkers of periodontitis identified to date in the general population [15,16], extracellular matrix metalloproteinases (MMPs), particularly the measurement of their active forms (aMMPs), have demonstrated significant potential [43]. MMPs concentrations are particularly elevated in the gingival crevicular fluid of DS individuals with gingivitis [44] and periodontitis [13], highlighting the need for future research to determine whether they represent an effective biomarker for the early development of destructive forms of periodontal disease in DS.

In the present study, we found no significant differences in genotype or allele distribution for the rs1126478 (A/G) marker between DS individuals with and without periodontitis nor between the DS group as a whole and the Bank controls. Previous studies have linked the rs1126478 polymorphism to periodontitis susceptibility in North American [45], African American [46], Taiwanese [47], and Japanese [48] populations. However, this association has not been observed in Caucasians [14,46], which may help explain our findings in the Caucasian DS cohort.

### Limitations

This study has several limitations that warrant cautious interpretation of the results. The study group was a convenience sample (all individuals with DS from the local geographic area were invited to participate), meaning no a priori sample size calculation was performed, and the statistical power of the results remains unknown. However, the sample size is larger than those in previous studies in the literature [21,44]. To minimize the examination time, periodontal diagnosis was based on Ramfjord index teeth. Although this index correlates well with full-mouth examination, it may underestimate periodontitis cases [49]. The absence of a control group without DS could provide insight into whether lactoferrin regulation differs systematically; however, this hypothesis has already been confirmed in a recent case-control study [29]. Other factors, such as oral hygiene habits, diet, and access to dental care, could significantly impact the results; however, this potential bias was minimized by enrolling individuals who were regular attendees at specific special education centers. Additionally, salivary lactoferrin levels can be elevated in individuals with active caries, a factor not considered in this study [50]. While the lactoferrin concentration was quantified in total saliva, its iron-binding capacity was not assessed. It has been shown that in certain types of periodontitis, lactoferrin levels may increase, but its iron saturation may decrease [51]. The allele frequencies (A and G) were similar when comparing DS individuals (A: 70.8%; G: 29.1%) to controls from the “National DNA Bank-Institute of Health Carlos III”, but differences greater than 5% were observed compared to data from the European repository (A: 65.1%; G: 34.9%) [52].

## 5. Conclusions

In conclusion, our findings indicate that in individuals with DS, there is no significant increase in salivary lactoferrin levels or in the lactoferrin/BCA ratio in the presence of periodontitis. Additionally, no significant genetic associations were found with the rs1126478 polymorphism. These results suggest that lactoferrin production in DS may not be upregulated in response to periodontal pathogens, which could be indicative of an immune system dysregulation contributing to the early onset and severity of periodontitis in this population. Further investigation, including the analysis of lactoferrin in gingival crevicular fluid and its iron saturation, is necessary to explore the functional implications of these findings.

## Figures and Tables

**Table 1:** Salivary lactoferrin, BCA, and lactoferrin/BCA ratio levels in individuals with Down syndrome based on periodontal health status.

	Periodontal Health (*n* = 13)	Gingivitis (*n* = 29)	Periodontitis (*n* = 34)	Kruskal–Wallis Test
**Lactoferrin (µg/mL)**				
Mean (sd)	8.73 (4.03)	7.90 (5.61)	7.83 (4.24)	H = 0.909 η^2^ = −0.016 *p* = 0.635
Median (Q1; Q3)	7.61 (5.23, 10.68)	6.57 (3.57, 10.21)	8.20 (4.88, 11.24)	
Range	4.56–18.57	0.84–20.37	0.21–14.74	
**BCA (µg/mL)**				
Mean (sd)	263.35 (193.15)	367.72 (253.70)	321.44 (235.78)	H = 1.893 η^2^ = −0.002 *p* = 0.388
Median (Q1; Q3)	208.14 (129.14, 348,71)	332.57 (171.86, 464.29)	261.89 (171.03, 369.57)	
Range	45.71–759.14	18.86–906.33	47.71–1015.50	
**Ratio (%)**				
Mean (sd)	5.09 (4.42)	2.83 (1.58)	3.72 (3.95)	H = 2.606 η^2^ = 0.009 *p* = 0.272
Median (Q1; Q3)	2.56 (2.36, 5.65)	2.64 (1.64, 3.87)	2.30 (1.41, 4.47)	
Range	1.92–15.92	0.26–6.36	0.13–6.72	

BCA: total protein concentration (bicinchoninic acid assay); ratio: lactoferrin/BCA; H: Kruskal–Wallis test statistic; η^2^: Kruskal–Wallis eta squared (effect size); *p*: *p*-value.

**Table 2:** Association of genotypes and alleles for the rs1126478 (A/G) marker in the study of CASES (Down syndrome individuals with periodontitis vs. Down syndrome individuals without periodontitis) and CASES vs. CONTROLS (Down syndrome individuals total vs. Bank controls).

	DS (CASES)				DS vs. Bank (CASES vs. CONTROLS)			
Genotype	DS PH/G *n* (%)	DS P *n* (%)	*p*-Value	OR (CI)	DS Total	Bank	*p*-Value	OR (CI)
**Codominant**								
AA	13 (50.0)	19 (55.8)	0.858 ^†^	1.00	32 (53.3)	529 (47.2)	0.633	1.00
GA	10 (38.4)	11 (32.3)		1.31 (0.50–3.47)	21 (35.0)	488 (43.6)		0.81 (0.52–1.26)
GG	3 (11.5)	4 (11.7)		1.17 (0.23–5.80)	7 (11.6)	103 (9.2)		0.97 (0.48–1.95)
**Allelic (log-Additive)**								
A	36 (69.2)	49 (72.0)	0.666	1.00	85 (70.8)	1565 (68.9)	0.591	1.00
G	16 (30.7)	19 (27.9)		1.16 (0.58–2.33)	35 (29.1)	705 (31.1)		0.92 (0.67–1.25)
**Recessive**								
AA + GA	23 (88.4)	30 (88.2)	0.955 ^†^	1.00	53 (88.3)	1017 (90.8)	0.852	1.00
GG	3 (11.5)	4 (11.7)		1.05 (0.22–4.96)	7 (11.6)	103 (9.2)		1.07 (0.55–2.08)
**Dominant**								
AA	13 (50.0)	19 (55.8)	0.593	1.00	32 (53.3)	529 (47.2)	0.409	1.00
GA + GG	13 (50.0)	15 (44.1)		1.28 (0.52–3.18)	28 (46.6)	591 (52.8)		0.84 (0.56–1.27)

DS: Down syndrome; PH/G: periodontal health/gingivitis; P: periodontitis; Bank: Spanish individuals with a healthy periodontal condition from the Spanish National DNA Biobank; ^†^ Fisher’s exact test was applied; OR: odds ratio adjusted for sex and age; CI: confidence interval.

## Data Availability

The original datasets are presented in the article. Further inquiries can be directed to the corresponding author.

## Supplementary Materials

The following supporting information can be downloaded at https://www.mdpi.com/article/10.3390/jcm14061815/s1: Figure S1: Flowchart of the study detailing the number of adults with Down syndrome, with and without periodontitis, in whom lactoferrin genotype (rs1126478 marker) and salivary concentration determinations were performed.

## Abbreviations

The following abbreviations are used in this manuscript:

DS	Down syndrome
A	Adenine
G	Guanine
BCA	Bicinchoninic acid (total protein concentration)
ELISA	Enzyme-linked immunosorbent assay
T/C	Thymine/cytosine
PH	Periodontal health
G	Gingivitis
P	Periodontitis
