# Supplementary material for: Salivary Lactoferrin Levels and Polymorphisms in Down Syndrome Individuals with Periodontitis
Source: J Clin Med. 2025 Mar 7;14(6):1815. doi: 10.3390/jcm14061815 (PMC11942906; doi:10.3390/jcm14061815)
Supplement: Supplementary file 1 [file jcm-14-01815-s001.zip › jcm-3472588-supplementary.pdf]

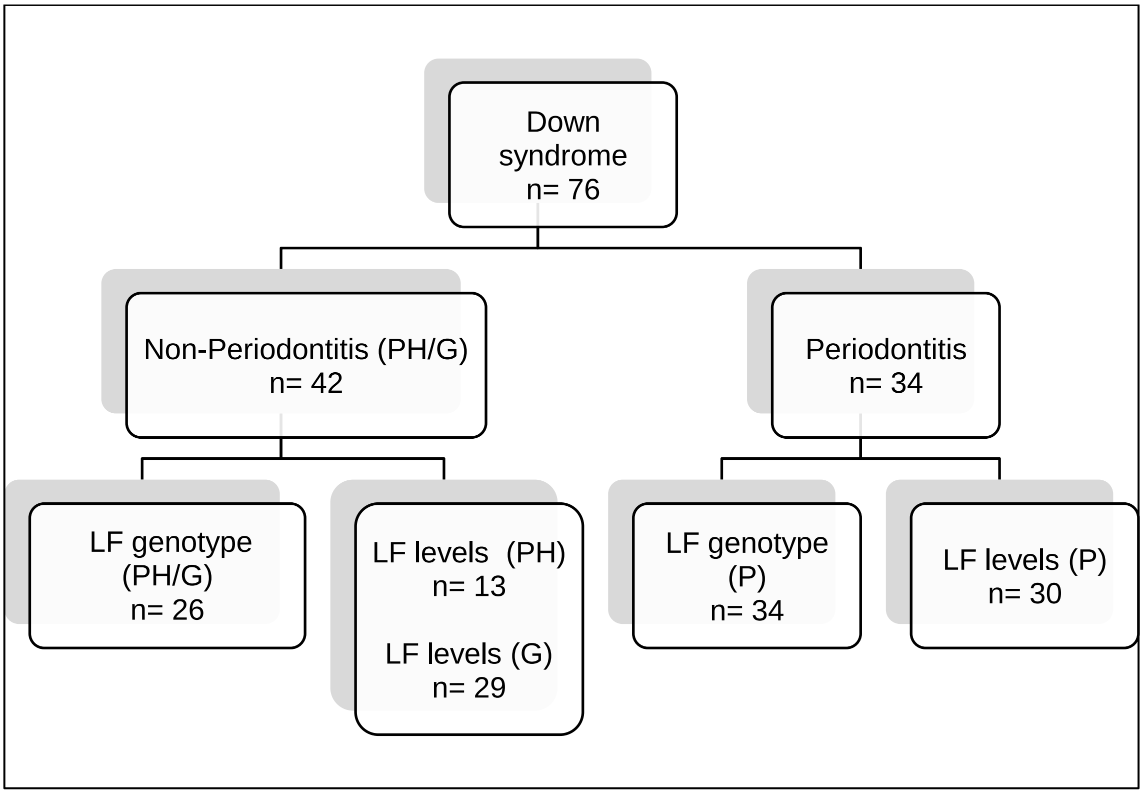


**Figure S1.** Flowchart of the study detailing the number of adults with Down syndrome, with and without periodontitis, in whom lactoferrin genotype (rs1126478 marker) and salivary concentration determinations were performed.

PH/G: periodontal health/gingivitis; P: periodontitis; LF: Lactoferrin; n= number of individuals.
